# Epigenetic nutrition in aquaculture: The emerging role of Artemia as a bioencapsulation vehicle

**DOI:** 10.1016/j.cirep.2026.200320

**Published:** 2026-09-30

**Authors:** Yathish Ramena, Arielle Shelby, Grace Ramena

**Affiliations:** a Department of Aquaculture and Fisheries, University of Arkansas at Pine Bluff, 1200 University Dr, Pine Bluff, AR, 71601, USA; b Aquaculture/Fisheries, Center of Excellence, University of Arkansas Pine Bluff, Pine Bluff, AR, 71601, USA; c Fish Inspection lab, University of Arkansas at Pine Bluff, 2001 Highway 70 East, Lonoke, AR, 72086, USA

**Keywords:** Artemia, Epigenetic regulation, Biovectors, Larviculture, Aquaculture nutrition

## Abstract

This review analyzes the emerging role of *Artemia*-mediated epigenetic regulation in aquaculture. *Artemia* is widely used as live feed for fish and shellfish larvae and can bioencapsulate nutrients, microorganisms, and other bioactive compounds, making it a potential delivery vehicle for candidate epigenetic-modifying compounds. Current studies demonstrate that Artemia can successfully deliver several candidate compounds, including poly-β-hydroxybutyrate, selenium, cobalt, choline-containing phospholipids, and probiotic-associated bioactive factors, with subsequent effects on growth, immunity, stress resistance, metabolism, and gene expression in recipient aquatic species. However, these physiological and transcriptional responses do not, on their own, establish epigenetic regulation. Mechanistic epigenetic links are primarily supported by the candidate compounds known bioactivity, which includes modulation of S-adenosylmethionine availability, histone deacetylase inhibition, and interactions with non-coding RNA. Demonstrations linking Artemia enrichment to recipient exposure, a defined epigenetic alteration, and the resulting phenotype remain limited.

Evidence for transgenerational epigenetic inheritance in aquaculture species following treatment with enriched Artemia remains unestablished. Future studies should focus on integrating *Artemia*-enrichment with directly measured epigenetic effects in larvae as wells as multigenertaional evaluations. Collectively, *Artemia* presents a promising platform for the delivery of candidate epigenators in aquaculture; however, stepwise validation is required to establish its ability to modulate epigenetic systems in aquaculture.

## Highlights

• Artemia offers an emerging platform for delivering candidate epigenetic modulators to aquaculture larvae.
• Nutritional programming during early development may influence growth, immunity, and stress-related phenotypes.
• Nutrients and bioactive compounds may influence DNA methylation, histone modification, and ncRNA pathways.
• Direct evidence linking Artemia-delivered compounds to defined epigenetic changes remains limited.
• Future studies should validate exposure, molecular mechanisms, causality, and transgenerational persistence.

## Introduction

*Artemia*, commonly known as brine shrimp, are a genus of microcrustaceans widely used as a live feed in aquaculture, particularly during the larval stages of fish and shellfish [1]. They naturally thrive in hypersaline environments but can remain viable in freshwater for a considerable length of time, permitting their use as a live feed for a diverse range of cultured species [1]. Because of their small size, broad environmental tolerance, and high nutritional content, *Artemia* are well-suited for supporting the growth of aquatic larvae [2]. Several studies have shown that larvae fed live *Artemia* have increased survival, higher growth rates, and improved appetite compared to larvae fed other live feeds [3]. These benefits may be further enhanced by their capacity for nutritional enrichment, through which *Artemia* function as a biological deliverer (biovector) of essential dietary compounds directly to the larvae [3]. Recently, research has suggested the potential to leverage *Artemia’*s enrichment capacity to deliver compounds capable of influencing epigenetic regulation in aquaculture species, which this review refers to as “epigenators” [4]. This approach could potentially modulate gene expression associated with improved growth, immunity, and long-term health in fish and shellfish [5].

The ability to manipulate epigenetic processes through dietary factors, termed “nutritional epigenetics”, has generated increasing interest in its use as a potential strategy for improving health and disease resistance in aquaculture species [6]. Globally, aquaculture loses an estimated 6 billion USD annually due to diseases, emphasizing the dire need for more effective disease control strategies [7]. Dietary compounds capable of influencing epigenetic processes may serve as candidate epigenators. Nutrition-based epigenetic modifications have already been studied in channel catfish, Nile tilapia, Pacific oyster, European sea bass, and several other relevant species, supporting further investigation into their viability as a disease-management strategy (Table 1) [8].

**Table 1:** Current epigenetic regulation applications in aquaculture species.

Species	Common Name	Developmental Stage & Initial Weight	Exposure / Intervention	Evaluated Phenotype	Measured Epigenetic Endpoint	Reference
*Litopenaeus vannamei*	Pacific white shrimp	Post-larvae (2.50 ± 0.01 mg)	PHB-enriched Artemia	Increased nutrient absorption, faster growth rate	Not measured	[9]
*Oreochromis niloticus*	Nile tilapia	Larvae, 10 dpf (no initial weight reported)	Temperature-dependent sex determination	Temperature-associated changes in *amh* expression correlated to a disproportionate % of males and females	Not measured	[10]
*Lates calcarifer*	Barramundi or Asian sea bass	Larvae, 11 dph (no initial weight reported)	Se-enriched live feed	Improved antioxidant defense, reduced skeletal deformities	Not measured	[11]
*Salmo salar*	Atlantic Salmon	Post-smolt (no initital weight reported)	Captive rearing / no experimental intervention	Initiation of precocious sexual maturation	Differential miRNA expression associated with precocious testiscular development	[12]

Note**.** poly-β-hydroxybutyrate (PHB); selenium (Se); omega-3 polyunsaturated fatty acid (n-3 PUFA).

Epigenetics encompasses changes in gene regulation that occur without altering the underlying DNA sequence [13,14]. These changes are often mediated at the chromatin or post-transcriptional level through mechanisms including histone modifications, DNA methylation, and non-coding RNA (ncRNA) activity [6]. Epigenetic regulation occurs naturally as a response to environmental stimuli but can also be experimentally induced in a controlled laboratory setting [15]. In the latter approach, known bioactive compounds may be administered to host species to direct these epigenetic changes [15].

Despite its potential, the application of epigenetics in aquaculture remains in its infancy. Epigenetics is limited by significant knowledge gaps, including the identification of canidate epigenators and the characterization of their associated molecular pathways [16]. Furthermore, the effective delivery of these compounds to early-stage larvae present a practical challenge due to the small size and concerns regarding epigenator stability and bioavailability [17]. Because *Artemia* are already routinely used as biovectors of nutrients, probiotics, and other bioactive compounds to aquatic larvae, it represents a promising potential delivery system for epigenators [18]. Therefore, this review aims to investigate the potential of *Artemia* as a biovector for epigenators and explore its viability as a health management and disease control strategy in aquaculture.

### Literature search and evidence evaluation

This review was developed through literature searches focused on *Artemia* enrichment, nutritional programming, and epigenetic regulation in aquaculture. Literature was primarily identified through a web-based search engine using terms that included “*Artemia* enrichment,” “*Artemia* bioencapsulation,” “aquaculture epigenetics,” “nutritional epigenetics,” “DNA methylation,” “histone modification,” “non-coding RNA,” “transgenerational epigenetic inheritance,” and the names of individual candidate epigenetic-modifying compounds discussed in this review. Additional studies were found from the references of these articles. Emphasis was placed on peer-reviewed studies in aquatic organisms and on studies directly evaluating *Artemia*-mediated delivery where available. Evidence from non-aquatic models was included when necessary to describe molecular mechanisms that were underdeveloped in aquatic animals.

Studies were evaluated based on the level of evidence provided. Studies that demonstrated bioencapsulation or delivery of candidate compounds, molecular or physiological responses following *Artemia*-mediated epigenator exposure were prioritized.

## *Artemia* as a nutritional delivery system in aquaculture

### Artemia usage in larviculture

*Artemia* have been used in larviculture operations since the early 1970s, when the Great Salt Lake emerged as a major commercial source of *Artemia* cysts [19]. Since then, *Artemia* have become one of the most widely-used live feeds in aquaculture, owing to their small size, commercial availability, and adaptability [3]*. Artemia* are typically acquired as dormant cysts that can be hatched under controlled conditions prior to larval feeding [20]. The newly hatched cyst, called a nauplius, is ideal for larval feeding due to its high moisture content, which eases larval digestion, as well as its capacity for nutritional enrichment [3]. Suitability is also influenced by characteristics of the larvae’s species, as mouth size, digestive capacity, and feeding behavior differ considerably among species [21]. Newly hatched nauplii are generally appropriate for smaller larvae, whereas larger *Artemia* stages may be better suited for larger or more developmentally advanced larvae [3]. Mortalities of up to 20 % have been observed in larvae fed overly large prey, emphasizing the importance of selecting adequately sized live feed in larviculture [22].

### Artemia enrichment strategies

Although *Artemia* provide several important nutrients, they are often deficient in key nutrients necessary for optimal larval development, particularly eicosapentaenoic acid (EPA) and docosahexaenoic acid (DHA) [23]. Both EPA and DHA are omega-3 polyunsaturated fatty acids (n-3 PUFAs) with essential roles during early larval development [23]. These n-3 PUFAs provide an important energy source for young larvae, as well as the structural components necessary for cell-membrane development [23]. DHA is especially important due to its high content in the nervous and retinal tissue of most fish [23]. Larvae lacking these n-3 PUFAs are suspected of having high mortality rates, visual and nervous system defects, suppressed immunity, and limited growth [24]. Because the larvae are unable to synthesize sufficient quantities of these key n-3 PUFAs, they must be dietarily provided [23]. As such, *Artemia* are frequently enriched with EPA, DHA, and other nutrients prior to their application in larviculture [25].

The enrichment capacity of *Artemia* is largely facilitated by the nauplii’s indiscriminate filter-feeder behavior, permitting nonselective ingestion of a diverse small particles [26]. During enrichment, particles containing the desired nutrient or bioactive compound are suspended in the water and subsequently engulfed by *Artemia* [26]. Following ingestion, the target nutrients bioaccumulate inside the *Artemia* and are transferred to the larvae upon consumption [27]. This process, known as bioencapsulation, utilizes *Artemia* as a living vector to deliver bioactive compounds to the larvae (Fig. 1) [28]. Organisms utilized in this manner are referred to as biovectors and encompass a wide range of species, including microalgae, bacteria, and fungi [29].

**Fig. 1:**
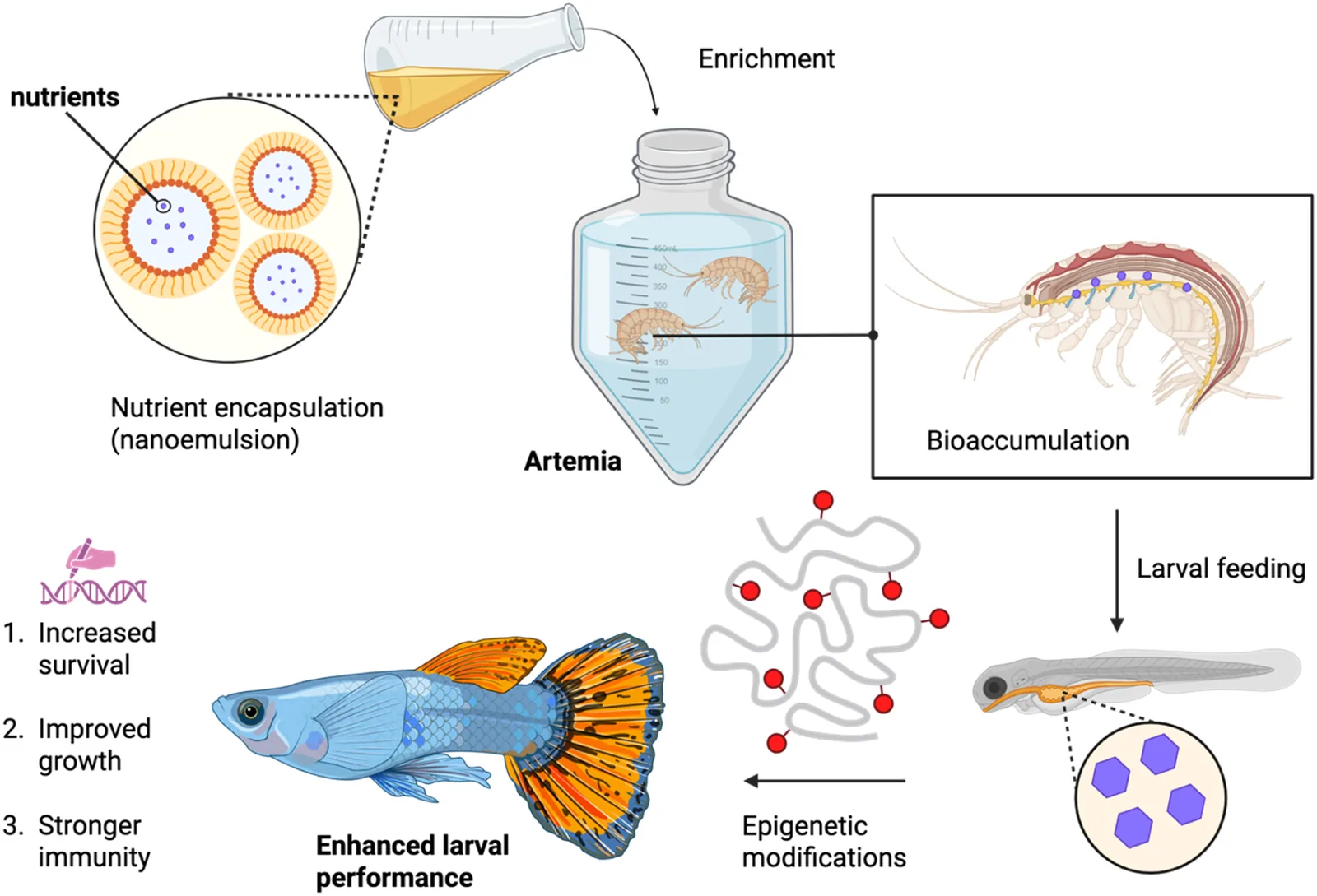
Illustration of the nutrient bioencapsulation process in *Artemia*.

Bioactive nutrients, probiotics, or epigenetically active compounds are incorporated into *Artemia* through bioencapsulation (enrichment). Following ingestion by the larvae, these compounds can influence molecular pathways involved in gene regulation, potentially altering physiological traits such as growth performance, immune function, and survival adaptability.

Successful *Artemia* enrichment and subsequent larval delivery depend on the stability of the target compound, its uptake and retention within *Artemia*, and bioavailability within the larvae after feeding. Following uptake, the compound may have several metabolic fates within the *Artemia*. It may accumulate within *Artemia* tissues, undergo metabolic transformation, or be excreted prior to larval consumption. Additionally, the compound must be released from *Artemia* tissues during larval digestion and reach its molecular target in a biologically active form. Effective delivery is therefore contingent upon the unique physiochemical properties of the molecule and the digestive and metabolic capacities of both the *Artemia* and recipient larvae.

### Nutritional programming

Many aquaculture species exhibit a high level of developmental plasticity in their early life, characterized by behavioral and physiological adaptations in response to environmental changes [30]. This plasticity generally decreases as the organism matures and becomes less sensitive to environmental cues [30]. Larviculture therefore provides a critical opportunity to modulate the long-term physiological and behavioral outcomes of aquaculture species in a process known as nutritional programming [31]. For example, *Artemia* nauplii supply their own digestive enzymes to the larvae upon being consumed, incidentally assisting in their own digestion and supporting the development of the larvae’s endogenous digestive system [32]. This effect is crucial during early larval development, as many larvae lack a formal digestive tract at this stage [32]. However, the contribution of digestive enzymes from the *Artemia* becomes less pronounced after the larvae reaches a juvenile stage, usually within 2–3 weeks when their digestive system is fully developed [33].

Nutritional programming may also negatively impact the health of aquaculture species [34]. In largemouth bass (LBM), diets containing greater than 25 % of soybean meal (SBM) was found to downregulate anti-inflammatory genes and suppress the expression of intestinal tight junctions, resulting in SBM-induced enteritis (SBMIE) [34]. LBM is known to be a high-mortality risk species, making them even more sensitive to inadequate nutrition [34]. Comparatively, *Litopenaeus vannamei* (Pacific White Shrimp) was shown to tolerate SBM levels up to 40 %, with marked intestinal morphology defects and nutritional malabsorption at levels exceeding this threshold [35]. When appropriately implemented, however, nutritional programming can promote favorable, and potentially transgenerational, phenotypic advancements. Nutritional epigenetic regulation presents one potential mechanism through which early nutritional exposure may produce changes in gene expression and phenotype. However, nutritional biological effects may also result from physiological and developmental processes independent of epigenetic regulation. This distinction is important when evaluating *Artemia*-mediated delivery of epigenators, as observed nutrition-based changes in physiology alone do not directly demonstrate an underlying epigenetic mechanism.

## Epigenetic regulation in aquaculture species

### DNA methylation

Epigenetic regulation involves multiple interacting mechanisms, including DNA methylation, histone and chromatin modifications, and ncRNA activity (Fig. 2) [36]. Collectively, these processes can alter gene regulation without changing the primary DNA sequence [37]. Of these mechanisms, DNA methylation is best characterized and involves covalent addition of a methyl group at the C-5 position on cytosine, forming 5-methylcytosine [38]. DNA methyltransferases, such as Dnmt1, Dnmt3a, and Dnmt3b, are the enzymes responsible for carrying out this process [38]. Dnmt3a and Dnmt3b primarily establish methylation *de novo,* while Dnmt1 maintains it, enabling patterns of methylation to persist through mitotic cell divisions [39].

**Fig. 2:**
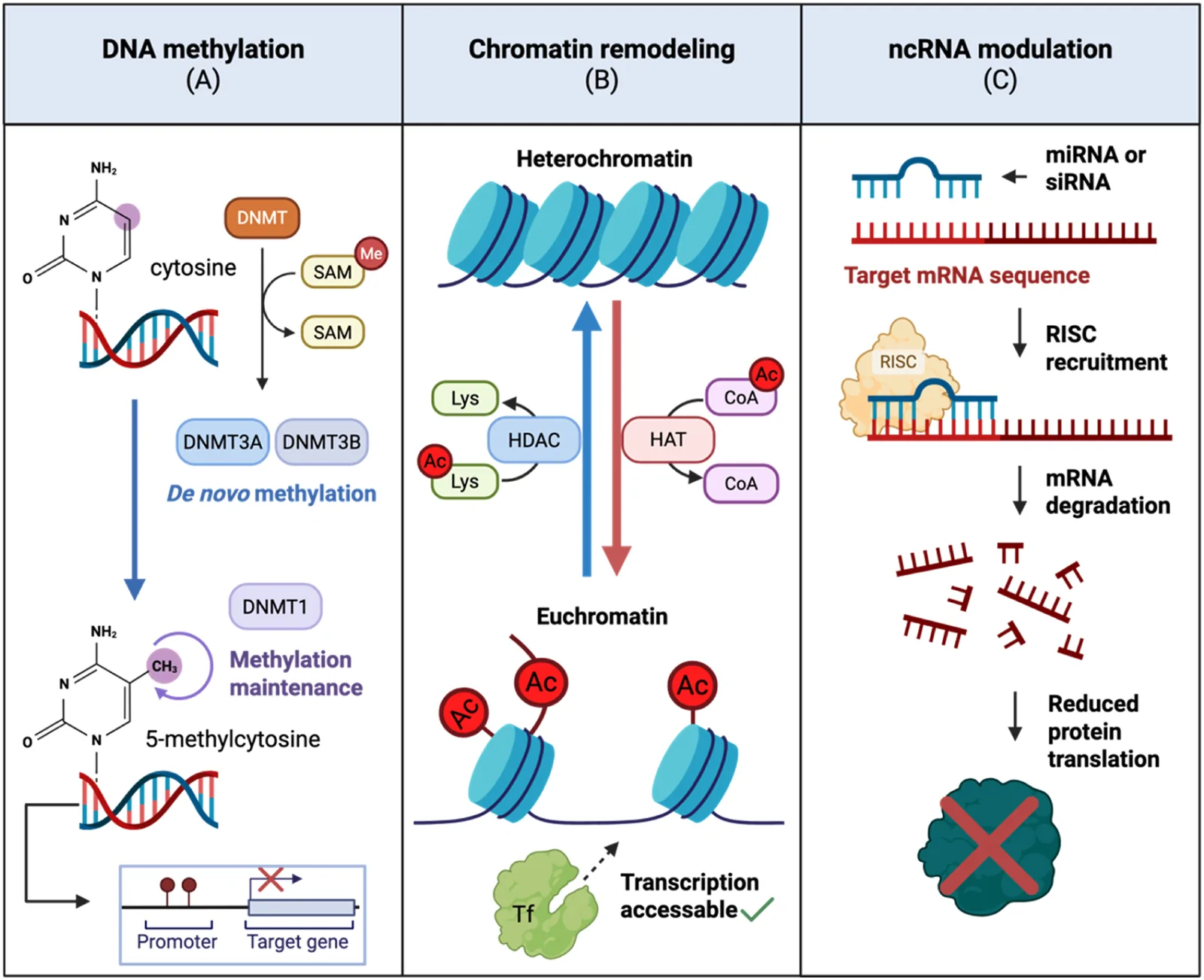
**Diagram of epigenetic mechanisms**. (A) DNA methylation mediated by DNA methyltransferase (Dnmt3a, Dnmt3b, Dnmt1). (B) Chromatin remodeling as a result of histone modifications featuring lysine acetylation and deacetylation by histone acetyltransferase (HAT) and histone deacetylase (HDAC) respectively. (C) Targeted mRNA degradation through ncRNA activity directed by RNA-induced silencing complex (RISC).

In aquatic vertebrates, DNA methylation is most frequent at CpG dinucleotides, although non-CpG methylation has been reported and may vary according to species, tissue, and developmental stage [40]. The effect of DNA methylation on gene regulation is largely context dependent [41]. When DNA methylation occurs at gene promoter sites, it is frequently associated with reduced transcription, potentially through interference with transcription-factor binding [42]. The capacity of gene silencing due to promoter methylation varies depending on the loci, cell type, and developmental stage [42]. This distinction is important when interpreting methylation changes in aquaculture species, where genome architecture and methylation patterns can differ considerably among taxa.

#### Histone acetylation and deacetylation

DNA methylation can also interact with chromatin-regulatory processes surrounding methylated CpG regions; however, whether these effects are mediated directly through histone interactions or indirectly through other pathways remains in question [43]. Histones are the major structural proteins involved in the organization of DNA into chromatin, in which DNA is wrapped around histone octamers to form nucleosomes [44].The nucleosome arrangement has a significant impact on gene regulation and expression. When nucleosomes are packed tightly together, the chromatin adopts a more condensed structure known as heterochromatin [44]. Heterochromatin is generally considered less transcriptionally accessible than euchromatin, in which the nucleosomes are further spaced apart [45]. These states, however, are dynamic and are regulated by various other chromosomal elements; therefore, euchromatin and heterochromatin should not be considered synonymous with transcriptionally “on” and “off” states [46].

Histone acetylation and deacetylation are heavily involved in chromatin regulation and are frequently associated with changes in transcriptional activity [47]. Histone acetylation occurs primarily at the lysine residues located along histone’s N-terminal tail and is mediated by histone acetyltransferase (HAT) [48]. Acetyl transfer neutralizes the lysine residue’s positive charge, weakening electrostatic interactions between histone proteins and DNA [48]. Disruption of these interactions is generally associated with looser chromatin structure and greater transcriptional accessibility [48]. Conversely, histone deacetylases (HDACs) remove the acetyl group, effectively restoring the lysine’s positive charge and reestablishing chromatin compaction [48].

Correlations between DNA methylation and histone regulation have been reported, including involvement of methyl-CpG-binding domain (MBD) proteins [49]. Certain MBD proteins can recognize methylated CpG regions and recruit chromatin-regulatory complexes containing HDACs to those sites, providing one mechanism through which DNA methylation may be associated with reduced chromatin accessibility and transcriptional repression [50]. Although DNA methylation patterns and histone modifications may be maintained across mitotic cell divisions, this level of persistence does not, by itself, establish germline or transgenerational inheritance [38].

#### ncRNA modulation

ncRNAs constitute a third layer of epigenetic regulation and refer to any RNA molecule that does not directly translate into a protein [51]. ncRNAs are categorized into several types, including microRNA (miRNA), small interfering RNA (siRNA), long non-coding-RNA (lncRNA) and Piwi-interacting RNA (piRNA) [52]. miRNAS are small ssRNAs ranging from 20–24 nt [52]. They are evolutionarily conserved across all eukaryotes and can inhibit mRNA translation of protein-coding genes [53]. Following transcription, miRNA is incorporated into an Argonaute-containing RNA-induced silencing complex (RISC), where it directs recognition of complementary sequences within target mRNA [53]. Depending on the extent of sequence complementarity and cellular context, miRNA binding can promote translational repression, transcript deadenylation and decay, and in some cases, direct cleavage of the miRNA [54]. Because target recognition does not require complete complementarity, an individual miRNA can target multiple transcripts and can thereby influence several pathways [54]. siRNAs function similarly but are usually generated from double-stranded RNA precursors [55]. RISC separates the ds-siRNA into single strands and utilizes one strand as a guide toward the complementary mRNA sequence, where cleavage is determined by the degree of sequence overlap [55]. lncRNAs may participate in broader epigenetic processes such as chromatin remodeling through proposed interaction with DNA and histone modifying enzymes like Dmnt3a and HDAC [56]. LncRNAs are typically longer than 200 nt and can recruit or scaffold chromatin-modifying complexes, thereby linking RNA activity to DNA methylation [56]. piRNAs primarily interact with PIWI-family proteins and are especially important for transposable-element suppression and genome integrity in germline cells [56]. Although their functions and mechanisms are diverse, ncRNAs collectively contribute to gene regulation and may participate in nutritionally responsive regulatory pathways in aquatic organisms.

### Nutritional factors in epigenetics

Many of the described epigenetic mechanisms begin with the reception of an environmental signal known as the epigenator [13]. Epigenators are a relatively new concept currently lacking a formal definition; the term is generally applied to any signal, either external and internal, that triggers the activity of an “epigenetic initiator” [13]. Epigenators can be chemical elements, like nutrients and bioactive compounds, or physical, such as temperature and physiological arousal [57]. It is well documented that changes in temperature can impact sex determination in some aquaculture species, occurring through alteration of anti-Mullerian hormone (*amh*) gene expression [58]. In another example, fish exposed to over-crowded conditions had changes in the DNA methylation patterns of autophagy genes as a result of prolonged cortisol production [59]. Physical factors such as stocking density and water quality can influence epigenetic regulation in aquaculture species; however, nutritional factors may provide a more controllable route for investigating targeted epigenetic modulation.

Nutritional influences on chromatin regulation have been investigated through several metabolic pathways. Several molecules can influence the activity of chromatin-modifying enzymes like Dnmt and HDAC through either direct inhibition or altering substrate availability [60]. For instance, folate contributes to DNA and histone methylation through its role in 1-carbon metabolism [61]. Folate mediates the production of *S*-adenosyl methionine (SAM), which serves as the primary methyl source for histone and DNA methylation [61]. Methionine and choline are also involved in SAM production, making them potential candidate nutrients for epigenetic modification [62]. However, most mechanistic studies on 1-carbon metabolites have centered on mammalian models, necessitating validation in aquatic vertebrates.

Besides methyl-donating compounds, the availability of acetyl-donating molecules may also potentially influence the rate and prevalence of epigenetic activity. Such molecules, like acetyl-coenzyme A (acetyl-CoA), are critical substrates for HAT-mediated histone acetylation [63]. HAT extracts the acetyl group from acetyl-CoA and transfers it to the lysine residues along the histone tail [63]. Multiple studies have documented the effect of intracellular acetyl-CoA concentration on the extent of histone acetylation, providing a putative link between nutrients that alter acetyl-CoA availability and epigenetic regulation [64].

Acetyl-CoA is synthesized as a product of glucose metabolism and fatty acid β-oxidation [65]. During glycolysis, pyruvate created from glucose is converted into acetyl-CoA by pyruvate dehydrogenase (PdH). In fatty acid β-oxidation, long-chain fatty acids like n-3 PUFAs are transformed into acyl-coenzyme A (acyl-CoA) by acyl-CoA synthetase and later undergo β-oxidation to produce successive units of acetyl-CoA depending on the length of the original fatty acid chain [66]. Diets high in lipids and carbohydrates have been shown to increase the intracellular concentration of acetyl-CoA in aquaculture species, warranting future investigation into their potential role in nutritionally mediated histone acetylation [67].

Probiotic organisms may also modulate epigenetic processes [68]. Probiotics alter gene expression by releasing bioactive metabolites and modifying ncRNA expression within the host [68]. Key metabolites produced by probiotics include short-chain fatty acids such as butyrate and acetate, which can function as HDAC inhibitors and acetyl-CoA precursors, respectively [69]. Probiotics have also been associated with the transference of exogenous miRNAs and modulation of host miRNA and lncRNAs, which may influence gene expression [68]. In aquaculture species, probiotic supplementation has been associated with phenotypic outcomes such as increased wound-healing, enhanced immune activity, and improved reproductive performance [70]. However, mechanistic evidence connecting probiotic metabolites to ncRNA regulation and other epigenetic processes is largely derived from human and non-aquatic models; therefore, evidence for the involvement of these mechanisms in aquaculture species remains indirect.

## Artemia as a delivery system for epigenetic regulation

### Artemia as biovectors for epigenators

As discussed previously, traditional *Artemia* enrichment strategies have focused on the bioencapsulation of n-3 PUFAs [3]. This strategy may potentially be applied to deliver compounds of putative epigenetic activity; however, their stability, uptake, and retention by *Artemia* are critical determinants of successful enrichment and subsequent delivery to larvae (Table 2). For example, in 2024, Kim et al. demonstrated the ability of *Artemia* nauplii to be enriched with poly-β-hydroxybutyrate (PHB) by adding dissolved PHB directly to the *Artemia* rearing tank [9]. PHB is a microbially-synthesized polymer that can be hydrolyzed to the short-chain fatty acid, β-hydroxybutyric acid (BHB or 3-HB), under physiological conditions [71,72]. The molecular pathway by which PHB is converted to BHB in aquatic animals has yet to be characterized; however, it is believed that certain bacteria within the gut microbiota have been proposed to facilitate this conversion through the release of PHB depolymerases (PhaZs) [71,73,74].

**Table 2:** Candidate epigenetic modulators delivered through *Artemia* or related biovectors and their proposed mechanisms.

Candidate Molecule	Delivery Route	Delivery Evidence / Phenotype	Proposed Epigenetic Mechanism	Evidence Limitations	Reference
**BHB**	Artemia	Altered growth; digestive/immune gene expression in *L. vannamei*	HDAC inhibition	BHB production/exposure and HDAC/histone changes not measured	[73]
**Selenium**	Artemia / rotifers	Improved growth/stress resistance; altered Se-associated gene expression	Methionine preservation for SAM-dependent DNA methylation	Indirect metabolic connection; DNA methylation not measured	[74]
**Cobalt**	Artemia	Increased growth and altered gene expression in *L. califer*	B12-dependent methionine production for SAM availability	SAM activity and DNA methylation not measured	[75]
**Probiotic Organisms**	Artemia / supplements	Host transcriptional/physiological responses	Changes to miRNA activity and expression; HDAC inhibition; substrate for HAT	Probiotic-miRNA modulation not studied in aquatic organims; HDAC activity not measured	[68,70,76]
**Folate**	Artemia	SAM-associated DNA and histone methylation	SAM synthesis	Direct DNA methylation not measured	[77]
**Methionine**	Artemia via liposomes	SAM-associated DNA and histone methylation	SAM synthesis	Direct DNA methylation not measured	[78]
**Choline**	Artemia via soybean letchin	Improved growh and enhanced lipid-catabolism gene expression	Supports methionine production for SAM availability	Choline contribution, SAM availability, and DNA methylation not measured	[79]

Note. *S*-adeonosyl-L-methionine (SAM); histone deacetylase (HDAC); glutathione peroxidase (GPX).

BHB has been shown to inhibit HDACs through proposed competitive enzyme inhibition, providing a potential mechanistic link to epigenetic regulation [80]. In a mouse model, treatment with BHB resulted in hyperacetylation of histones in kidney cells that is consistent with direct HDAC inhibition [80,81]. Furthermore, in vitro treatment of a human embryonic kidney cell line (HEK293) with BHB led to increased acetylation at FOXO3a and Mt2 promoters, consequently upregulating these genes [80]. These findings provide evidence that BHB-mediated HDAC inhibition can promote histone acetylation at specific promoters, allowing for increased expression of targeted genes. The extent to which this mechanism extends to epigenetic regulation in aquatic animals remains unclear however, as very few studies have reported direct evidence of BHB-mediated HDAC inhibition in aquaculture species. Moreover, BHB is not necessarily the most potent inhibitor of HDAC, as butyrate has demonstrated stronger HDAC-inhibitory activity in some studies [82].

Given the evidence that BHB can influence gene expression through HDAC inhibition, the potential for *Artemia* to deliver PHB as a precursor of BHB to aquaculture species has recently been investigated. Kim et al. evaluated the impact of feeding PHB-enriched *Artemia* to *L. vannamei* [9]. When delivered to *L. vannamei* using *Artemia* nauplii as a vehicle, genes associated with nutrient absorption, particularly trypsin, chymotrypsin, and amylase, were significantly upregulated compared to a control [9]. Expression of growth regulatory genes such as insulin-like growth factor (IGF-1) and its cognate transporter, insulin-like growth factor binding protein (IGF-BP), was also upregulated in *L. vannamei*, leading to a faster growth rate [9]. PHB-enriched *Artemia* also exerted immunomodulatory effects on *L. vannamei* by enhancing the expression of prophenoloxidase (proPO), a precursor for phenoloxidase (PO) that can encapsulate pathogens and increase the rate of wound-healing [9]. Elevated PO activity was correlated with increased *Vibrio parahaemolyticus* resistance in *L. vannamei* [9]. Although these findings establish that PHB-enriched Artemia can alter gene expression and metabolic responses in Pacific white shrimp, this study did not demonstrate whether HDAC inhibition or changes in histone modification were the mechanisms behind these observations. Therefore, BHB-mediated HDAC inhibition represents one plausible mechanism underlying the observed upregulation of digestive and immune-associated genes, but further research is required to experimentally validate this connection.

Because of its reported HDAC-inhibitory activity, BHB represents a potential class of epigenators that may alter epigenetic enzyme activity directly. Other candidate epigenators may influence these processes indirectly by effecting the availability of substrates or cofactors required for epigenetic enzyme activity. Such compounds include methionine, choline, folate, and long-chain fatty acids that were introduced in the preceding section, as well as selenium and cobalt [11,83].

Choline contributes to 1-carbon metabolism by its conversion to betaine, which acts as an alternative methyl-source for the production of methionine—ultimately supporting SAM formation [84]. Choline can be released from phosphatidylcholine through the action of phospholipases in aquatic animals, a major phospholipid component in the eukaryotic cell membrane [85]. As a supplement, phosphatidylcholine is typically derived from soybean lecithin, which contains a variety of phospholipids [86]. Because of choline’s supportive role in SAM production, soybean lecithin-derived phosphatidylcholine represents a potential epigenator source in aquaculture. Soybean lecithin has already been used in *Artemia* enrichment, and *Nibea albiflora* (yellow drum) larvae exhibited improved growth and enhanced lipid catabolism gene expression when fed lecithin-enriched *Artemia* [79]. However, these findings do not establish that choline or phosphatidylcholine was responsible for the observed effects, nor do they directly demonstrate changes in SAM availability or epigenetic regulation.

Selenium (Se) is a critical component of the selenocysteine (Sec) catalytic center of glutathione peroxidase (GPX) [74]. GPX is responsible for converting toxic hydrogen peroxide (H_2_O_2_) and organic hydroperoxides into water or their corresponding alcohol counterpart [74]. This is necessary to prevent the accumulation of reactive oxygen species (ROS) known for causing oxidative stress in aquatic animals [87]. In 2022, Pham et al. demonstrated that Asian sea bass larvae fed with selenium-enriched *Artemia* had less skeletal deformities, improved stress resistance, and faster growth [11]. Regarding its impact on gene expression, other selenium-necessitating proteins, particularly *msrb1* (an oxidized methionine recycler) and *sep15* (a recycler of oxidized thioredoxins) were significantly upregulated in Atlantic bluefin tuna larvae fed with Se-enriched rotifers [88]. Because Msrb1 contributes to the recovery of oxidatively damaged methionine, its upregulation could assist in preserving the methionine pool for SAM production. Selenium-dependent oxidative protection may therefore indirectly contribute to the maintenance of cellular capacity for epigenetic regulation.

Similarly, cobalt (Co) may also influence 1-carbon metabolism through its role as an essential component of vitamin B12, which functions as a necessary cofactor for methionine synthesis—thereby supporting SAM-dependent methyltransferase activity [75,89]. Co is incorporated into the central corrin ring of vitamin B12, which facilitates the transfer of a methyl group from 5-methyltetrahydrofolate to homocysteine during methionine production [90]. This interaction provides a plausible biochemical connection between cobalt nutrition, SAM availability, and DNA methylation; however, whether Co supplementation effects DNA methylation patterns directly in aquatic species remains unclear.

To evaluate the effects of Co supplementation in aquatic livestock, Fehér et al. fed barramundi larvae *Artemia* nauplii enriched with either 50 or 100 mg/L CoCl_2_ [83]. Upon enrichment, Co concentrations were significantly higher in the nauplii and correspondingly in the barramundi larvae following predation—demonstrating the viability of *Artemia* as a vehicle for Co [83]. Larvae fed Co-enriched *Artemia* exhibited considerable growth improvement and enhanced expression of antioxidant and immune-related genes following predation compared to a control; however, these effects were highly dose-dependent due to the toxicity potential of cobalt [83]. Although no toxicity was reported in the barramundi larvae at these levels, larvae fed the 50 mg/L Co-enriched *Artemia* exhibited a greater growth improvement than those fed the 100 mg/L Co-enriched *Artemia*; indicating that optimization of cobalt enrichment levels is required to avoid diminishing returns in larval development [83]. Moreover, although *Artemia*-based Co supplementation had positive physiological and nutritional effects on the larvae, there was no direct evidence demonstrating that epigenetic regulation mediated the observed responses.

Beyond nutrient bioencapsulation, *Artemia* can also be enriched with probiotic bacteria capable of producing bioactive metabolites with potential epigenetic activity [3]. In this approach, Artemia serve as the primary delivery vehicle for the probiotic organism, which may provide an additional source of bioactive compounds through the production of bacterial metabolites. Diets of probiotic-enriched *Artemia* have been associated with increased pathogen resistance and growth rate in aquatic animals, with the most commonly used species being of the *Bacillus* and *Lactobacillus* genera [3]. These effects are generally attributed to mechanisms including modification of the intestinal microbiota, competitive exclusion of pathogens, immune stimulation, and production of bioactive microbial metabolites [68]. Probiotic supplementation has also been associated with changes in host gene expression and corresponding physiological phenotypes in fish [70]. For example, supplementation of *Pediococcus acidilactici* in male zebrafish was correlated to increased transcription of reproductive-related genes, including *bdnf, dmrt1, lepa*, and *fshb*, resulting in improved sperm motility and greater reproductive performance [70]. Moreover, some metabolites produced by bacteria have the potential to interact with host epigenetic pathways directly. For instance, the probiotic *Clostridium butyricum* produces the well-established HDAC inhibitor butyrate through the fermentation of dietary carbohydrates, providing a potential link between probiotic activity and histone regulation [76,91]. When introduced to Pacific white shrimp, *C. butryicum* was associated with enhanced expression of immune-related genes, including *CTL, proPO, Crustin*, and *ALF1–3*, as well as increased intestinal concentration of butyrate and other short-chain fatty acids [76]. Yet despite these apparent benefits, whether probiotic-enriched *Artemia* can modulate epigenetic mechanisms in aquaculture species remains unexplored.

### Transgenerational implications

Epigenetic modifications induced by environmental or nutritional exposures may, in some cases, persist across generations [92,93]. If reliably induced and maintained, such responses could have important implications for aquaculture by extending beneficial phenotypes beyond the initially treated generation and potentially reducing the need for repeated intervention. However, not all epigenetic modifications are stable or transmissible across generations, and subsequent environmental exposures may alter or reverse previously established epigenetic states [94]. Tracing epigenetic modifications across progeny is therefore a challenging endeavor and is further complicated by the necessity of distinguishing intergenerational from transgenerational inheritance [92,93].

Transgenerational inheritance refers to the persistence of an exposure-associated epigenetic state of phenotype observed in generations that were not exposed to the original epigenator [92,93]. Consequently, epigenetic inheritance may be considered transgenerational when these effects persist in several generations beyond the exposed parent [92,93]. In contrast, intergenerational inheritance refers to epigenetic modifications that are generally observed in the direct descendants of the exposed parent [92,93]. These descendants are presumed to have also faced exposure to the epigenator during development, which is made apparent by the disappearance of the trait in subsequential generations when exposure is removed Multigenerational inheritance encompasses both intergenerational and transgenerational inheritance and is generally described as epigenetic modifications that persist across multiple generations regardless of exposure to the epigenator [92,93].

The generation at which an effect can be considered transgenerational depends on the epigenator exposure route, sex of the exposed parent, and life cycle of the species. In live-bearing aquaculture species, maternal F0 exposure can directly expose the F1 embryos during development and consequentially the germ cells that will give rise to the F2 generation (Fig. 3) [95]. For this reason, transgenerational studies in live-bearing species are generally assesed at the F3 generation and beyond if the maternal parent received treatment [96]. Conversely, if the paternal parent received treatment, only the F1 generation is most likely to be exposed through the sperm during fertilization, allowing the F2 generation to be considered for transgenerational inheritance [97]. Likewise in oviparous species, transgenerational studies may also be conducted at the F2 generation, as the F1 generation can be exposed through the unfertilized egg or sperm [98].

**Fig. 3:**
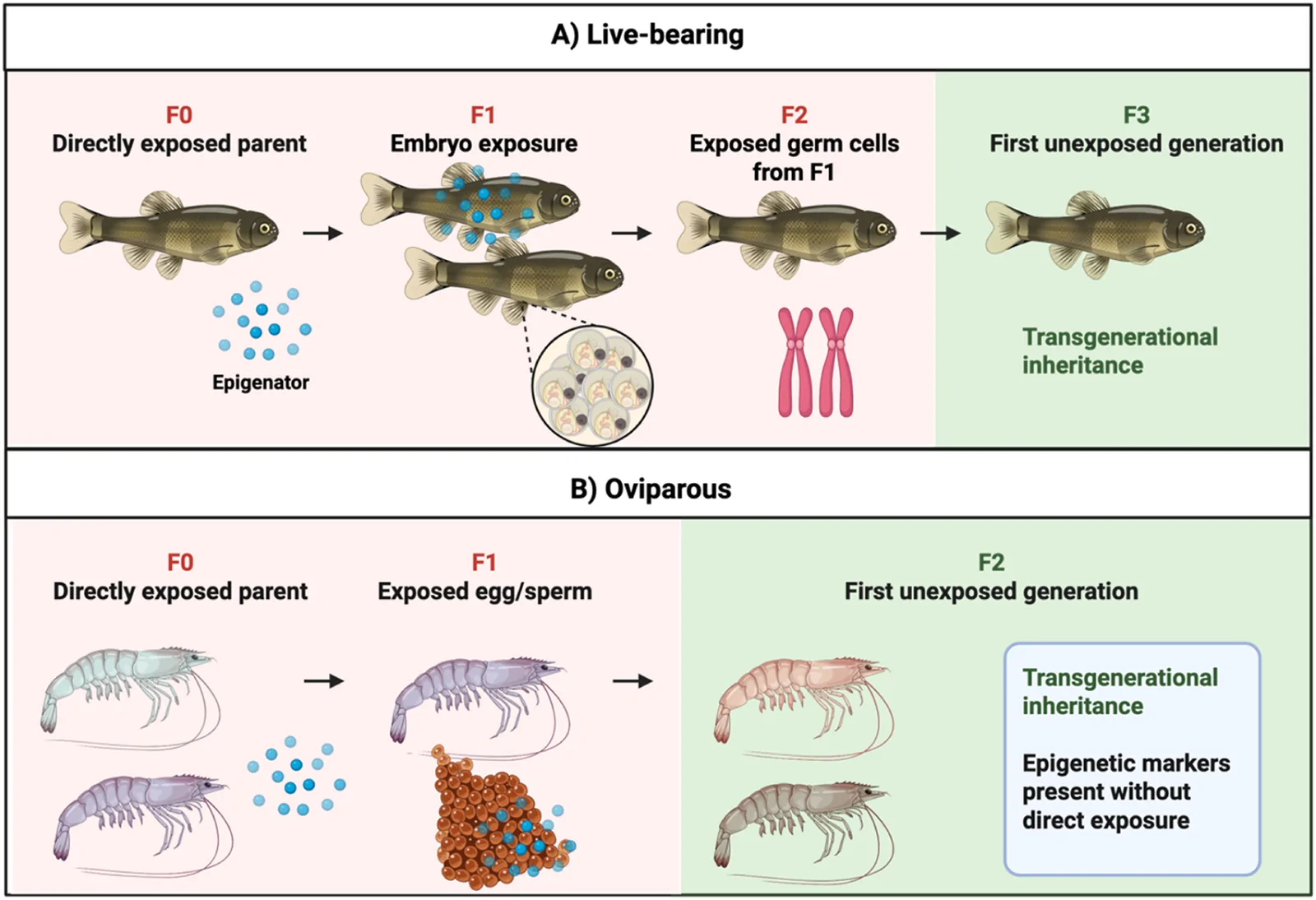
**Multigenerational and transgenerational epigenetic inheritance showing F0, F1, F2, and F3 generations.** (A) In live-bearing species, transgenerational inheritance studies begin during the F3 generation as F1 offspring are directly exposed to the epigenator during embryo development and the F2 offspring is exposed from the inherited germ cells of F1. (B) In oviparous (egg-laying) species, transgenerational epigenetic inheritance can be traced earlier in the F2 generation as only the F1 offspring are directly exposed to the epigenator as an unfertilized egg or pre-ejaculated sperm.

There are several studies in aquatic organisms that demonstrate epigenetic-associated phenotypes that persist in offspring beyond the directly exposed generation [99]. In 2022, Bautista et al. investigated the changes in DNA methylation patterns for zebrafish housed in crude oil-contaminated water and found a significant decrease in the global DNA methylation for those exposed to crude-oil and also in their offspring that were not exposed; however, the effects were generally found to persist only into the F2 generation, suggesting intergenerational inheritance [100]. In another study, Poecilia mexicana (Atlantic molly) exposed to toxic hydrogen sulfide (H_2_S) exhibited distinct DNA methylation patterns on genes associated with sulfur metabolism and toxicity that overlapped with more than 80 % of the methylation patterns in the unexposed F2 generation, demonstrating potential transgenerational inheritance [101]. Furthermore, phloroglucinol treatment of *Artemia* increased the expression of innate immune genes and resistance to *V. parahaemolyticu*s and *V. Harveyi* which persisted into the F3 generation [102]. Similarly, in *Artemia* subjected to nonlethal heat shock, in which the F0 generation exhibited increased heat tolerance and higher resistance to *Vibrio campbelli* that was later seen in F1, F2, and F3 generations [103].These findings are particularly relevant for the use of *Artemia* as an epigenator biovector; however, phenotypic expression alone does not establish an epigenetic mechanism.

### Molecular approaches to assess epigenetic regulation

The evaluation of epigenetic regulation in aquaculture species relies on molecular and bioinformatic approaches that determine changes in DNA methylation, histone modification, and ncRNA [36]. These approaches can be used to identify molecular changes associated with epigenator exposure and assess whether these changes persist across generations. Whole-genome bisulfite sequencing (WGBS) is a reliable technique for analyzing DNA methylation patterns in which bisulfite is used to convert unmethylated cytosines into uracil, while 5-methylcytosine is unaffected [104]. Sequencing and alignment of the bisulfite-treated DNA allows the methylated and unmethylated cytosines to be distinguished throughout the genome, which could reveal differentially methylated regions associated with epigenator exposure [104]. However, the accuracy of WGBS may be diminished by incomplete bisulfite conversion, sequencing depth, read mapping, and cell-type heterogeneity [105]. Furthermore, WGBS cannot predict whether changes in DNA methylation are responsible for altered gene expression or phenotypic representation [106].

Chromatin immunoprecipitation followed by sequencing (ChIP-seq) can be used to investigate changes in histone modifications [107]. ChIP-seq utilizes antibodies to target specific histone modifications, allowing identification of genomic regions enriched with histone acetylation [107]. However, ChIP-seq requires a relatively large amount of DNA and is limited by false-positive signals arising from antibody promiscuity and DNA-protein crosslinking [107,108].

Analyzing the expression and novelty of ncRNAs may be accomplished through high-throughput RNA sequencing (RNA-seq) and quantitative real-time polymerase chain reaction (RT-qPCR) [36]. Small-RNA sequencing is useful for profiling shorter ncRNAs like miRNAs, whereas total-RNA sequencing can provide more global transcriptomic information, including longer ncRNAs [104]. Following identification of the ncRNA’s sequence, RT-qPCR can be used to validate its expression [109]. Therefore, combining ncRNA profiling with DNA methylation, histone modification, and gene-expression analyses may help identify relationships between epigenetic regulation and phenotypic responses across generations. However, changes in ncRNA abundance, histone modifications, chromatin accessibility, or DNA methylation alone do not establish a causal relationship with changes in gene expression or phenotype and instead require careful integration with physiological measurements and functional validation.

## Challenges and limitations

Epigenetic applications in aquaculture remain limited by experimental, biological, and genomic factors. Although relatively stable, DNA methylation is highly reversible, complicating its use as a long-term epigenetic marker [110]. Furthermore, characterization and interpretation of DNA methylation and histone modification patterns are heavily dependent on the availability and quality of whole-genome assemblies, which are incomplete or lacking in several important aquaculture species [36]. Additionally, epigenetic mechanisms can differ greatly between species, limiting the extent to which findings from one species can be extrapolated to another, further highlighting the necessity of acquiring high-quality genome sequences for all aquaculture species [36].

Interpretation of DNA methylation and histone modification is also complicated by their endogenous, universal roles in normal development and gene regulation [111]. As a result, distinguishing environmentally induced epigenetic processes from baseline gene regulation necessitates a high level of variable control that may not always be practical, especially in vivo studies. Factors including changes in water temperature, salinity, dissolved gas levels, and random physiological events within the organism may influence experiment results and restrict data reproducibility. In studies on epigenetic inheritance, it epigenetic modulating source during transgenerational studies or fluctuating environmental states must be considered during experimentation. Certain variables, like random physiological events, cannot be controlled and are capable of undermining results and restricting experimental reproducibility.

Regarding its commercial applicability, there is no guarantee that a proposed epigenator will produce the intended molecular or phenotypic response, nor do so consistently. Epigenetic responses can vary according to dose, developmental stage, tissue, environmental conditions, and interactions with other regulatory processes. Transposable elements (TE), which are mobile genetic elements capable of changing loci and directing their own amplification, can occur spontaneously and result in new genetic alterations that override the changes made by the epigenator [5]. Furthermore, the *Artemia* may exhaust or chemically alter the epigenator during their own metabolic processes, preventing successful delivery to larvae. Some epigenator sources may also negatively impact gene regulation in aquaculture species when administered in inadequate dosages or in harmful combination with other genetic modulating compounds. For example, both cobalt and n3-PUFAs were shown to stunt growth performance and increase oxidative stress when administered to larvae in excess [112,113]. Dose optimization and toxicological assessment are therefore necessary before these compounds can be considered for practical application.

### Delivery validation and exposure quantification

A major limitation in the use of *Artemia* as a biovector is the difficulty of determining successful bioenrichment and effective delivery to larvae. Observing the candidate epigenator within the enriched *Artemia* indicates uptake, but does not establish that an effective dose reaches the target tissues of the recipient larvae. Each stage of the delivery pathway therefore, requires experimental validation to establish dose-response relationships between the concentration supplied in the enrichment medium, the concentration accumulated within the *Artemia*, and the concentration reaching the larvae. This is supported by the differences reported in barramundi larvae fed 50 mg/L of Co-enriched *Artemia* and 100 mg/L Co-enriched *Artemia* [83]*.* The chemical form and distribution of the compound within the *Artemia*, as the larvae could receive a functionally altered or inactive version of the compound. Upon ingestion, histological analysis may be necessary to verify the presence and quantity of the epigenator within the larvae; however, this could require excisions that result in termination of the larvae and the inability to view the effects of the epigenator post-larvae. However, without these controls, larval outcomes cannot be direct attributed to the proposed epigenator.

## Future directions

Future efforts in *Artemia*-mediated epigenetic modulation should prioritize validation of the complete pathway from enrichment to the observed phenotype. An effective experimental workflow requires accurate determination of epigenator loading concentration within enrichment media and *Artemia* after uptake*,* followed by precise measurements in larval tissue. Upon standardization of delivery procedures, studies must then shift toward qualitative characterization of the corresponding phenotype through histone modification and DNA methylation profiling, as well as RNA-seq for ncRNA determination. Establishing a causal relationship will require stringent control over variables and functional perturbation of the proposed regulatory system, including genetic inhibition, knockdown, and supplementation experiments. Access to high-quality genomic references will be essential for the design and interpretation of such experiments; therefore, efforts should be directed at assembling whole-genome sequences. For example, several species of Indian major carp, such as *Cirrhinus mrigala* (mrigalo), and certain catfish like *Clarias macrocephalus* (bighead catfish) lack or have incomplete genome assemblies [114]. The major obstacles that limit inclusive whole-genome sequencing in aquaculture species include high sequence repetition, extreme individual variability (heterozygosity), and whole-genome duplication (polyploidy) [115]. Many of these obstacles can be overcome by advancements in long-read sequencing (LRS) technology, which can analyze longer segments of DNA at a time, resulting in reduced system processing errors arising from repetition and paralogous genome duplication [116]. Significant progress has already been made on the whole-genome assembly of several aquaculture species utilizing LRS [[117], [118]].

Further investigations into intergenerational and transgenerational inheritance patterns will require rigorous variable isolation. Husbandry factors such as temperature, salinity, dissolved oxygen (O_2_), and feed rate must be standardized across generations to reduce confounding effects. Furthermore, experimental designs must distinguish direct exposure of developing offspring or germ cells from persistence of epigenetic modifications that emerge in generations not directly exposed. Longitudinal measurement of the relevant molecular endpoint across developmental stages and generations will therefore be necessary to establish sufficient evidence for transgenerational inheritance.

## Conclusion

*Artemia* has been a staple live feed in aquaculture for several decades due to its impressive nutritional content, larval digestibility, and enrichment capacity. These notable qualities have garnered attention for *Artemia*’s use as a biovector for epigenators. Epigenetic modulation has been shown to increase pathogen resistance, growth rate, and stress responses in aquaculture species, increasing the survival rate of young larvae. Bioactive compounds linked to epigenetic pathways involving methyltransferases, acetyl and deacetylation, and production of essential vitamins have been shown to enhance the abundance and activity of these enzymes. Furthermore, beyond their inherent microbiome-modulating effects, probiotics have additionally been shown to upregulate and even secrete ncRNAs that can alter the activity of specific genes. Many of these epigenetic regulation mechanisms have already been applied in aquaculture; however, their large-scale adoption in the industry has been limited by challenges in delivery efficiency, dosage optimization, acquisition of high-quality whole-genome sequences, and shortcomings of bioinformatics. The advancement of LRS technology, demarcation of epigenator safety profiles, improved bioencapsulation techniques, and replicable design of transgenerational inheritance studies will be necessary for the future of *Artemia*-delivered epigenator compounds to improve the health and viability of aquaculture species.

## Ethics declaration

The author(s) declare(s) that the study does not involve humans nor animal subjects.

## CRediT authorship contribution statement

**Yathish Ramena:** Validation, Project administration, Funding acquisition, Formal analysis, Data curation, Conceptualization. **Arielle Shelby:** Writing – review & editing, Writing – original draft, Formal analysis, Data curation. **Grace Ramena:** Resources, Methodology, Investigation, Funding acquisition, Formal analysis, Data curation, Conceptualization.

## Declaration of competing interest

The authors declare that they have no known competing financial interests or personal relationships that could have appeared to influence the work reported in this paper.

## Data Availability

Data will be made available on request.
